# Entry and Disposition of Zika Virus Immune Complexes in a Tissue Culture Model of the Maternal-Fetal Interface

**DOI:** 10.3390/vaccines9020145

**Published:** 2021-02-11

**Authors:** Yanqun Xu, Yong He, Sanaz Momben-Abolfath, Nancy Eller, Malgorzata Norton, Pei Zhang, Dorothy Scott, Evi Budo Struble

**Affiliations:** Laboratory of Plasma Derivatives, Division of Plasma Protein Therapeutics, Office of Tissues and Advanced Therapies, Center for Biologics Evaluation and Research, U.S. Food and Drug Administration, Silver Spring, MD 20993-0002, USA; Yanqun.Xu@fda.hhs.gov (Y.X.); Yong.He@fda.hhs.gov (Y.H.); Sanaz.MombenAbolfath@fda.hhs.gov (S.M.-A.); nancy.eller@fda.hhs.gov (N.E.); Malgorzata.Norton@fda.hhs.gov (M.N.); Pei.Zhang@fda.hhs.gov (P.Z.); Dorothy.Scott@fda.hhs.gov (D.S.)

**Keywords:** anti-Zika antibodies, anti-Zika IgG, antibody dependent enhancement

## Abstract

Zika virus (ZIKV) infections have been associated with an increased incidence of severe microcephaly and other neurodevelopmental disorders in newborn babies. Passive immunization with anti-ZIKV neutralizing antibodies has the potential to become a feasible treatment or prophylaxis option during pregnancy. Prior to clinical use, such antibodies should be assessed for their ability to block ZIKV passage to the fetus. We used human placental and mammalian cell monolayers that express FcRn and laboratory preparations of anti-ZIKV antibodies as a model system to investigate the disposition of ZIKV/antibody immune complexes (ICs) at the maternal-fetal interface. We further characterized solution properties of the ICs to evaluate whether these are related to in vitro effects. We found that both ZIKV and ZIKV envelope glycoprotein can enter and passage through epithelial cells, especially those that overexpress FcRn. In the presence of ZIKV antibodies, Zika virus entry was bimodal, with reduced entry at the lowest (0.3–3 ng/mL) and highest (µg/mL) antibody concentrations. Intermediate concentrations attenuated inhibition or enhanced viral entry. With respect to anti-ZIKV antibodies, we found that their degradation was accelerated when presented as ICs containing increased amounts of ZIKV immunogen. Of the two monoclonal antibodies tested, the preparation with higher aggregation also exhibited higher degradation. Our studies confirm that intact Zika virus and its envelope immunogen have the potential to enter and be transferred across placental and other epithelial cells that express FcRn. Presence of anti-ZIKV IgG antibodies can either block or enhance cellular entry, with the antibody concentration playing a complex role in this process. Physicochemical properties of IgG antibodies can influence their degradation in vitro.

## 1. Introduction

Zika virus is associated with ~100-fold increased incidence of severe microcephaly and other neurodevelopmental disorders in newborn babies especially when infection occurs during the first trimester [1,2,3]. It is believed that, like other arboviruses, ZIKV elicits a robust protective immune response, making the development of a vaccine highly desirable. However, vaccine trials in the face of an waning epidemic have proven difficult [4]. Prophylactic or therapeutic passive immunization with anti-ZIKV immune globulin G (IgG) antibodies, including during pregnancy, has been proposed and tested as an option [ClinicalTrials.gov studies NCT03624946 (IGIV), NCT03443830 (monoclonal antibody)].

Compared to other endogenous serum proteins, IgG molecules, including monoclonal and polyclonal antibody therapies, have a long half-life, ranging from 11–30 days [5]. The molecular basis of this phenomenon is the interaction between the neonatal Fc receptor (FcRn) and the Fc domain of the antibody. FcRn, a heterodimeric membrane bound receptor expressed in most mammalian cells interacts with IgG and albumin in a pH-dependent manner [6]. This interaction, which takes place inside acidified endosomes, prevents endosomal degradation and mediates release of IgG molecules into the lumen of blood vessels. The half-life of IgG is decreased (and clearance increased) when the target immunogen is present. It is postulated that Fc gamma (Fcγ) receptors in immune cells play a role in this expedited clearance [5]. Studies to assess the contributions of FcRn other receptors in this clearance have not been performed.

Human IgG antibodies are transported across the placenta during pregnancy by an active process mediated by the FcRn [7]. The maternal-to-fetal antibody transfer can provide protection from infections acquired both in utero and in the early post-natal period. Some viruses, in complexes with antibodies, exploit this essential pathway to cross epithelial [7] or placental [8] barriers, leading to fetal infection and disease. Recently, it was proposed that ZIKV antibodies may play a role in placental infection by SARS-CoV-2 [8], by amplifying infection of placental resident immune (Hofbauer) cells [9]. It is not known if anti-ZIKV antibodies can similarly enhance infection of placental trophoblast or mediate maternal-to-fetal passage of ZIKV through the FcRn antibody transport pathway.

Here we report the results of experiments measuring disposition of ZIKV IC in two mammalian cell lines that express high levels of FcRn. One is an MDCK cell line overexpressing human FcRn [10], often used as a model to study FcRn-mediated processes in an epithelial polarized monolayer. The other is a syncytium forming human choriocarcinoma cell line [11], a commonly used model of the syncytiotrophoblast, the placental structure separating the fetus from the maternal circulation. Using these cell lines, we found that intact ZIKV or the soluble fragment of ZIKV envelope glycoprotein (gpE) can enter and passage through these epithelial cell monolayers. The presence of monoclonal antibodies can either inhibit or enhance cellular entry, depending on their concentration and the cell line used. Furthermore, antibodies degrade more quickly in both cell lines when presented as ICs, compared to when there is no immunogen present. The rate of degradation could be related to the physical-chemical properties of these antibodies, such as the levels of protein aggregation in solution.

## 2. Methods

### 2.1. Zika Glycoprotein E (gpE) Immunogen and Zika Virus

Soluble, histidine-tagged recombinant gpE, derived from African strain and expressed in insect cells were obtained commercially (Meridian Life Sciences, Memphis, TN, USA).

Zika virus (Puerto Rican strain) used in this study was isolated by CDC from the serum of a ZIKV infected patient who travelled to Puerto Rico in 2015. The complete genome sequence is published (PRVABC59, Gene bank KU501215). Infectious virus was grown in Vero E6 cells (ATCC) after inoculation at low multiplicity of infection (MOI, 0.2–0.4, for the initial virus preparation obtained from CDC) in MEM media supplemented with 2% FBS. Then the virus was purified based on a published protocol [12]. Briefly, after 3–9 days (based on cytopathic effect) supernatant was harvested after centrifugation at 4000× *g* for 15 min. Virus was concentrated by precipitating overnight with 40% PEG 8000 in NTE buffer (10 mM Tris-HCl, pH 8.0, 120 mM NaCl and 1 mM EDTA) at a final concentration of 8% PEG then centrifuged at 14000× *g* for 30 min. The pellet was resuspended in NTE buffer and next purified with 25% sucrose cushioning and centrifugation at 30,000× *g* for 1.5 h. All the purification steps were performed at 4 °C. The final titer was determined via a standard plaque (TCID_50_) assay in Vero E6 cells.

### 2.2. Antibodies

The following polyclonal sera and monoclonal immune globulin G antibodies (IgG) were purchased from commercial sources. ZENV14-M (mAb14) and ZENV17-M (mAb17) were purchased from Alpha Diagnostic International (San Antonio, TX, USA). mAb14 is a human monoclonal IgG1 anti-ZIKV gpE domain III that does not cross-react with Dengue or other flaviviruses and was reported to enhance ZIKV infection in K562 cells via Fc gamma receptor (product data sheet). mAb17 is a humanized monoclonal IgG1 anti-flavivirus envelope protein domain II that cross-reacts with other flaviviruses. Purified myeloma IgG1 (Kappa) from human plasma was purchased from Sigma (catalog number I5154-1MG) and used as unrelated control for viral entry experiments.

The following antibodies were used for western blots. To detect gpE: mouse-anti-ZIKV gpE (1:5000, BF-1176-56, BioFront Technologies Sarasota, FL, USA), rabbit-anti-ZIKV gpE (1:1000, ZENV11-A, Alpha Diagnostic International), and donkey-anti-mouse IgG conjugated to HRP (1:50000, A90-337P, Bethyl Laboratories Inc., Montgomery, TX, USA); to detect human IgG: goat-anti-human Fc conjugated to HRP (1:20,000, A80-304P, Bethyl Laboratories Inc., Montgomery, TX, USA).

### 2.3. Cells

BeWo (human choriocarcinoma cell line) clone b30 was provided by Erik Rytting (University of Texas Medical Branch) and Marvin Darby Canine Kidney Cell line 2 (MDCK2) transfected with human FcRn receptor was provided by Richard Blumberg (Harvard University). The cells were passaged (less than 30 passages) in DMEM Glumax^®^ supplemented with 10% fetal bovine serum (FBS), non-essential amino acids (NEAA) and Antibiotic-Antimycotic mixture (AA, Thermo Fisher Scientific, Pittsburgh, PA, USA).

Vero E6 cells (ATTC) were used to grow and titer ZIKV. They were passaged in MEM medium supplemented with 10% FBS (2% FBS in experiments with ZIKV), NEAA and AA as above.

### 2.4. Assessment of Transcytosis of Immune Complexes (IC) Across Epithelial Cell Layers

**Figure 1:**
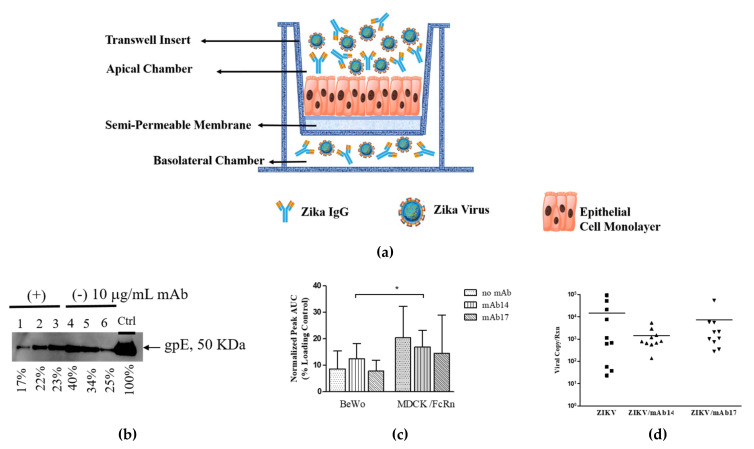
Transcytosis of Zika virus envelope glycoprotein (gpE) or intact Zika virus alone or as part of immune complexes (IC) in epithelial cell monolayers. (**a**) Schematic of the experimental set up. MDCK cells that overexpress human FcRn and BeWo cells were grown on semipermeable membranes; IC were added on the apical chamber. (**b**) Typical Western blot analysis of the contents of the basolateral chamber of transcytosis experiments with MDCK/FcRn or BeWo cells, blotting for gpE. Shown are three replicates for each IC performed with 10:100 (lanes 1–3) and 0:100 (lanes 4–6) µg/mL IgG:gpE mixtures; experiment was repeated to confirm the results. Signal intensities for each band were computed using densitometry (ImageJ) and are presented as a percentage of the positive control. Uncropped image of the Western blot is shown in Supplemental Figure S1. (**c**) Quantification of band intensity from western blots such as the one shown in (**b**) using densitometry (ImageJ). Each column represents the average signal from five or six replicates; error bars represent standard deviation. Two-way ANOVA was used to compare MDCK/FcRn (shaded bars) and BeWo (clear bars) cells (row factor); * *p* < 0.05. (**d**) Transcytosis of intact Zika virus in MDCK/FcRn and BeWo cell monolayers occurs for the virus alone or in the presence of mAb. Experiments were performed in triplicates or quadruplicates and repeated three times. Shown are the repeats from experiments performed in MDCK/FcRn cells. Similar high variability but lower group averages was seen in BeWo cells.

Single cell suspensions of MDCK/FcRn (seeding density 1.5 × 10^4^ per well) or BeWo (seeding density 1.5 × 10^5^ per well) cells were grown in transwell semi-permeable membranes (6.5 mm insert, 0.4 µm pore size, 0.33 cm^2^ cell growth area) placed into 24 well plates (Corning, Sigma-Aldrich, St. Louis, MOUSA) in replicates of three per test. Cell growth was monitored by measuring trans epithelial electrical resistance (TEER) via EVOM2 voltohmmeter (World Precision Instruments, Sarasota, FL, USA). When TEER attained 200–250 Ohm for BeWo and 400–450 Ohm for MDCK/FcRn, usually on day 4, the transcytosis experiment was performed. For this gpE alone (10 and 100 µg/mL), IgG-gpE (1:10 and 1:1 weight ratios) IC, or IgG alone (10 and 100 µg/mL) in 0.2 mL pre-warmed Hanks Balanced Salt Solution (HBSS) pH 6.0 were added inside the transwell; 0.2 mL HBSS pH 7.4 was added outside the transwell. IgG-ZIKV IC were generated by mixing 5x10^5^ TCID_50_ ZIKV (approximate multiplicity of 5 TCID_50_ per cell) with 10 µg/mL IgG antibodies inside the transwell, as before. A schematic of the experiment is shown in Figure 1.

After incubation for 90–120 min at 37 °C, buffer in the basolateral chamber was collected and assessed for the presence of gpE (western blot), ZIKV (PCR), and IgG (ELISA). The wells with increased volume in the basolateral chamber were excluded from measurement due to apparent paracellular transport.

To assess transcytosis of intact ZIKV, viral RNA was extracted from the basolateral buffer using QIAamp^®^ Viral RNA Kit (www.qiagen.com) following the manufacturer’s instructions. Ten µL of RNA was used as template for RT qPCR. To assess cell-associated IC, cells attached to the membrane were washed 1× with PBS, then lysed with 60 µL 1× NUPAGE LDS sample buffer with reducing agent and loaded into denaturing gel for Western blots. Gels were run using SDS under denaturing conditions (NuPAGE 4–12% Bis-Tris Protein Gels, Thermo Fisher Scientific, Pittsburgh, PA, USA), transferred onto a nitrocellulose mini membrane using iBlot system (Invitrogen) then assessed for the presence of either gpE or IgG using primary and secondary antibodies (see *Antibodies*). The same denaturing gels and Western blotting procedure were used to assess gpE transcytosis. A fixed amount of mAb or gpE was loaded in all the gels to serve as an internal normalizer for quantification (see *Data Processing and Statistical Analysis* for more details). A commercial kit (ELISA Kit, EI7200-1, AssayPro, St. Charles, MO, USA) was used to quantitate antibody transcytosis. The experiment was repeated on a different day to confirm the results.

### 2.5. Assessment of ZIKV IC Cell Entry

Viral entry assays were performed as reported in literature [13], with the modification that crude cell lysates (instead of purified RNA) were used for PCR analysis. Immune complexes were prepared in triplicate by mixing 10 µL virus with 10 µL IgG solution or dilution media (2% FBS in DMEM) and incubated on ice for approximately 1 h. An unrelated IgG1 antibody purified from human myeloma (Sigma-Aldrich, St. Louis, MO, USA) was used as negative control. Pre-formed IC or ZIKV alone were next added onto 5 × 10^5^ MDCK/FcRn or BeWo cells (approximate multiplicity of 100 TCID_50_ per cell) and incubated at 37 °C for one hour. Viral entry was halted by placing the cells on ice, followed by washing with cold PBS. The plates were next incubated over ice with Protease K (500 ng/mL) (Promega) for one hour, to digest any residual ZIKV attached to cell surfaces. Cells were washed three times with cold PBS and cell pellets were lysed with 200 uL iScript buffer (Bio-Rad) following the manufacturer’s instructions. The lysate was centrifugated at 20,000× *g* for three minutes, and supernatants were transferred into fresh tubes. These supernatants were diluted 1:100. RT-qPCR was performed on 4 uL aliquots of each sample, for ZIKV and GAPDH (see below). The experiment was repeated on a different day to confirm results (unrelated IgG controls were run in triplicate, one time only).

### 2.6. PCR

To assess the amount of ZIKV in purified RNA (transcytosis experiments) or crude cell lysates, one-step quantitative RT PCR was used (TaqMan^TM^ RNA-to-CT^TM^ 1-Step Kit from Applied Biosystems, www.thermofisher.com); primers were chosen to allow for high sensitivity as reported [14], and were synthesized by Integrated DNA Technologies, Inc. (www.idtdna.com). Each 50 or 10 uL PCR reaction solution contained 0.5 µM primer, 0.25 µM probe and RT-PCR master mix master as per manufacturer’s instructions. The PCR reaction and analysis were performed in a QuantStudio3 Q-PCR instrument and software from Applied Biosystems (www.Thermofisher.com). All samples were run in duplicate.

For transcytosis experiments the levels of ZIKV RNA were quantified using a standard curve, prepared using well-characterized material produced at FDA and harmonized during testing for the development of the WHO International Standard for ZIKV RNA [15]. Purified RNA from concentrated and titrated ZIKV was used to construct the standard curve. For the viral entry experiments ZIKV RNA was quantified using ∆∆C_t_ method, with GAPDH as the endogenous control (housekeeping) gene. Control wells containing ZIKV alone were used to establish infection baselines in the absence of IgG, Table 1.

### 2.7. Characterization of IC

#### 2.7.1. Surface Plasmon Resonance (SPR) (Biacore)

The SPR experiments were performed on a Biacore T200 instrument (Cytiva, Marlborough, MA, USA). A Human Antibody Capture Kit (Cytiva) was used to capture both mAb14 and mAb17 onto a CM5 chip (Cytiva). Briefly, the anti-human IgG (Fc) antibody provided at 0.5 mg/mL in 0.15 M NaCl was diluted to 25 µg/mL in Immobilization buffer (10 mM sodium acetate pH 5.0). Immobilization of antibody onto the CM5 chip was performed using an Amine Coupling Kit (Cytiva) and each following step was performed at 5 µL/min for 7 min. Three flow cells (Fc1, Fc2, and Fc3) of a CM5 chip (Cytiva) were activated with a 1:1 mixture of 1-ethyl-3-(3-dimethylaminopropyl)carbodiimide hydrochloride (EDC) and N-hydroxysuccinimide (NHS) after which the diluted anti-human IgG (Fc) was immobilized saturating the surface at 13,300–14,200 RU. The surface was deactivated using 1.0 M ethanolamine-HCl pH 8.5. Following immobilization of the capture antibody, mAb14 and mAb17 were diluted to 3 µg/mL in 0.01 M HEPES pH 7.4, 0.15 M NaCl, 0.005% *v*/*v* Surfactant P20 (HBS-P) and captured on Fc2 and Fc3, respectively, for 40 sec. at a flow rate of 10 µL/min to reach approximately 150 RU, aiming for a Rmax of 50 RU. Fc1 was kept blank as a reference surface for buffer subtraction (capture antibody only). Glycoprotein E was diluted to 200 nM starting concentration in HBS-P and then in 1:2 dilutions (200–3.125 nM) was run over each captured mAb and reference surface at a flow rate of 50 µL/min for 5 min after running 3 startup cycles with HBS-P buffer only over all the surfaces under the same conditions. The mAbs were allowed to dissociate for 10 min after which the surface was regenerated down to the anti-human Fc antibody using 3M magnesium chloride at a 20 µL/min flow rate for 30 s. The resulting sensograms were analyzed using in Biacore Insight Evaluation Software and fit using a 1:1 Langmuir model to obtain kinetics and affinity data.

#### 2.7.2. Dynamic Light Scattering (DLS)

Aggregation of biologic preparations can be assessed by measuring hydrodynamic diameters, which estimates the size and distribution of sizes of particles in solution. A Zetasizer Nano ZS (Malvern Instruments Ltd., Westborough, MA, USA) DLS instrument with 173° detection optics was used to measure estimate the size and size distribution of mAb14, mAb17, gpE, and their 1:1 molar mixtures. The samples were analyzed using a standard protein protocol with the material refractive index (RI) set at 1.45. The dispersant parameters were set based on the RI and viscosity of PBS, which were 1.330 and 0.8882, respectively. The measurement position and attenuator settings were automatically determined by the instrument. Three measurements were performed on each sample.

### 2.8. Data Processing and Statistical Analysis

ELISA readings were transformed into human IgG concentrations using a five-parameter fit of the standard curve (SoftMax Pro, Molecular Devices, San Jose, CA, USA). The concentration was expressed as a percentage of the IgG concentration added to the input chamber and analyzed using 2-way ANOVA with GraphPad Prism 5 (GraphPad Software, Inc., San Diego, CA, USA) with ICs as the column factor and mAb14 or 17 as the row factor; Bonferroni post-hoc analysis was used to correct for multiple comparisons.

Western blots were scanned using GBOX Mini (Syngene, syngene.com) imaging system. The image files were then analyzed with image processing software ImageJ (open source, version 1.52a). The bands for gpE or mAb were selected and plotted, then the area under the density peak curve (AUC) for each band was calculated using the software tools. Experimental AUCs were divided by the AUC of concurrent loading controls to allow for comparisons between multiple experiments. The differences in normalized AUCs was analyzed with 2-way ANOVA, with Bonferroni correction using GraphPad Prism 5.

ZIKV cellular entry in the presence or absence of mAb was quantified using ∆∆C_t_ method with GAPDH as the internal control. Briefly, human or canine GAPDH levels were used to calculate ∆C_t_ for BeWo and MDCK/FcRn cells, respectively. ∆C_t_ values from the experiments with ZIKV alone were used as the baseline to calculate ∆∆C_t_. Difference in cellular ZIKV RNA in the presence versus absence of mAb was then calculated using the expression: Fold Change = 2^(−∆∆Ct)^. The data set was analyzed with 2-way ANOVA as before using GraphPad Prism 5; *p* < 0.05 was considered significant.

## 3. Results

### 3.1. Assessment of Transcytosis of IC Across Epithelial Cell Layers

Transcytosis experiments were performed to assess whether the presence of anti-ZIKV antibodies would have an effect on the ability of intact ZIKV or its major immunogen, envelope glycoprotein to traverse epithelial cell monolayers. We found that Zika virus envelope glycoprotein, when added inside transwells containing confluent monolayers of BeWo or MDCK/FcRn cells at a concentration of 100 µg/mL (~1 µM dimer), can be found in the basolateral chambers as detected by Western blot (Figure 1b). A lower concentration of gpE did not result in appreciable transcytosis as detected by this method (data not shown). For both cell lines and both antibodies tested, the addition of IgG in the input chamber did not significantly change the amount of gpE in the output buffer (Figure 1b,c). Compared to MDCK/FcRn cells, transcytosis of gpE was significantly lower in BeWo cells (Figure 1c).

**Figure 2:**
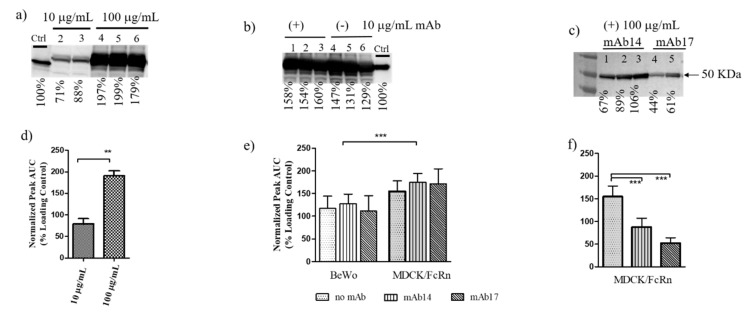
Cell association of Zika virus envelope glycoprotein immunogen (gpE) alone or as part of immune complexes (IC) in epithelial cell monolayers. (**a**–**c**) Typical western blot analysis of the contents of the MDCK/FcRn or BeWo cell monolayer of transwell assays; blotting for gpE. (**a**) Experiments with 10 (lanes 2–3) and 100 (lanes 4–6) µg/mL gpE in MDCK/FcRn performed in two and three independent repeats, respectively (shown). (**b**) Experiments with 10:100 (lanes 1–3) and 0:100 µg/mL (lanes 4–6) IgG:gpE mixtures; each experiment was performed in triplicate and repeated to confirm the results in both cell lines. (**c**) Experiments with 100:100 µg/mL IgG:gpE; mAb14 lanes 1–3 and mAb17 lanes 4–5. Experiment was performed in two or three repeats in MDCK/FcRn cells only. Signal intensities for each band were computed using densitometry (ImageJ) and are presented under each image as a percentage of the positive control. Uncropped images of the western blots are shown in Supplemental Figure S1. (**d**–**f**) Quantification of band intensity from Western blots such as those shown in (**a**–**c**) respectively using densitometry (ImageJ). Each column represents average values from (**d**,**f**) 2–3 and (**e**) 5–6 repeats; error bars represent standard deviation. (**d**) t-test was used to compare cell association of 10 and 100 µg/mL gpE; ** *p* < 0.01. (**e**) Two-way ANOVA was used to compare MDCK/FcRn and BeWo cells (row factor) and absence/presence of IgG (column factor); *** *p* < 0.001. (**f**) One-way ANOVA with Bonferroni correction was used to compare the means, *p* < 0.001. Note that “no mAb” bar for MDCK/FcRn cells is the same for (**e**,**f**).

In addition to probing the basolateral chamber for the presence of gpE in the exit chamber, we also collected and analyzed the cellular monolayers to assess the retention of the immunogen inside cells. GpE could be detected in monolayer lysates for both cell lines including at the lowest concentration tested, 10 µg/mL (Figure 2a,d). The addition of sub-stochiometric IgG in the input chamber did not affect the amount of gpE associated with cell monolayer (Figure 2b,e). Like gpE transcytosis, cell association of gpE was significantly lower in BeWo cells than MDCK/FcRn cells (Figure 2e). The addition of mAb14 and 17 at approximate stochiometric parity between mAb and gpE (100:100 µg/mL, or 1.5:1 uM concentration) resulted in a significant decrease in the amount of cell-associated (internalized) gpE (Figure 2c,f). Similar results were also seen at 10:10 µg/mL IgG:gpE ratio (data not shown). The same ratios were not tested in BeWo cells, given the lower levels of transcytosis and cell association observed in these cells.

Immune complexes between intact virus and IgG were also generated by mixing 5 × 10^5^ TCID_50_ ZIKV with 10 µg/mL antibody solution inside the transwell, followed by transcytosis analysis in MDCK/FcRn and BeWo cells, as before. The output chamber was assessed for the presence of viral RNA; the results are shown in Figure 1d. ZIKV RNA was detected at the basolateral chamber irrespective of the presence of IgG, indicating transcytosis. There was no difference in the transcytosis of ZIKV in the presence and absence of the antibodies, however, the individual measurements displayed a large degree of variability. Similar highly variable results were seen with BeWo cells (data not shown). As it was the case for gpE, the levels of intact ZIKV transcytosed by BeWo cells were lower than MDCK/FcRn cells.

**Figure 3:**
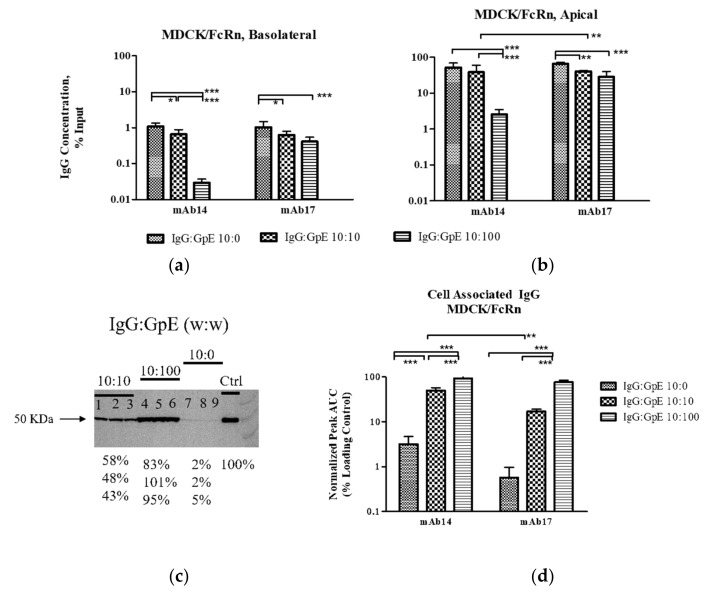
Transcytosis, internalization and degradation of IgG-gpE immune complexes (IC), assaying for IgG. IC were formed by mixing IgG and gpE at nominal w:w ratios of 10:0, 10:10 and 10:100 µg/mL in the apical chamber of transwells containing monolayers of MDCK/FcRn or BeWo cells. Average data from three independent repeats are shown, error bars represent standard deviation. (**a**) Less transcytosis and (**b**) lower residual levels of IgG were seen with increasing amounts of antigen in the basolateral and apical chambers, respectively (data from experiments with MDCK/FcRn cells, analogous results were obtained from BeWo cells). (**c**) Representative western blot of the contents of the cell monolayer lysate; probing for IgG heavy chain. Each lane contains an independent repeat, three repeats for each IC are shown. (**d**) Quantification of band density using ImageJ shows cell retention of IgG increasing with increased gpE concentration. Signal intensities for each replicate are presented as a percentage of the positive control at the bottom of the western blot image. Uncropped image of the western blot is shown in the Supplemental Figure S1. * *p* < 0.05, ** *p* < 0.01, *** *p* < 0.001.

One of the goals of our research was to evaluate the disposition of the IgG portion of the immunogen-antibody ICs. To this end, in addition to measuring cell retention/passage of the antigen, we evaluated transcytosis, internalization and degradation of the antibodies (Figure 3). Transcytosis and remaining (residual) levels of IgG in MDCK/FcRn cells were assayed at the basolateral (Figure 3a) and apical (Figure 3b) chambers, respectively for mAb14 and 17 using ELISA, whereas the internalized IgG was assayed using Western blots (Figure 3c,d). Less transcytosis and lower residual IgG in the output and input chambers, respectively were seen with increasing amount of antigen in both cell lines, suggesting increased degradation of IgG. Coincidentally, western blots of the cell monolayer lysate show increased cellular retention of IgG as antigen concentration was increased (Figure 3c; quantification in Figure 3d). Similar results were seen in BeWo cells (data not shown).

At the highest gpE concentration, mAb14 undergoes more robust degradation than mAb17, as indicated by significantly lower amounts of residual IgG in the input chamber and less transcytosis in the basolateral output (Figure 3a,b).

### 3.2. Assessment of ZIKV IC Cell Entry

**Figure 4:**
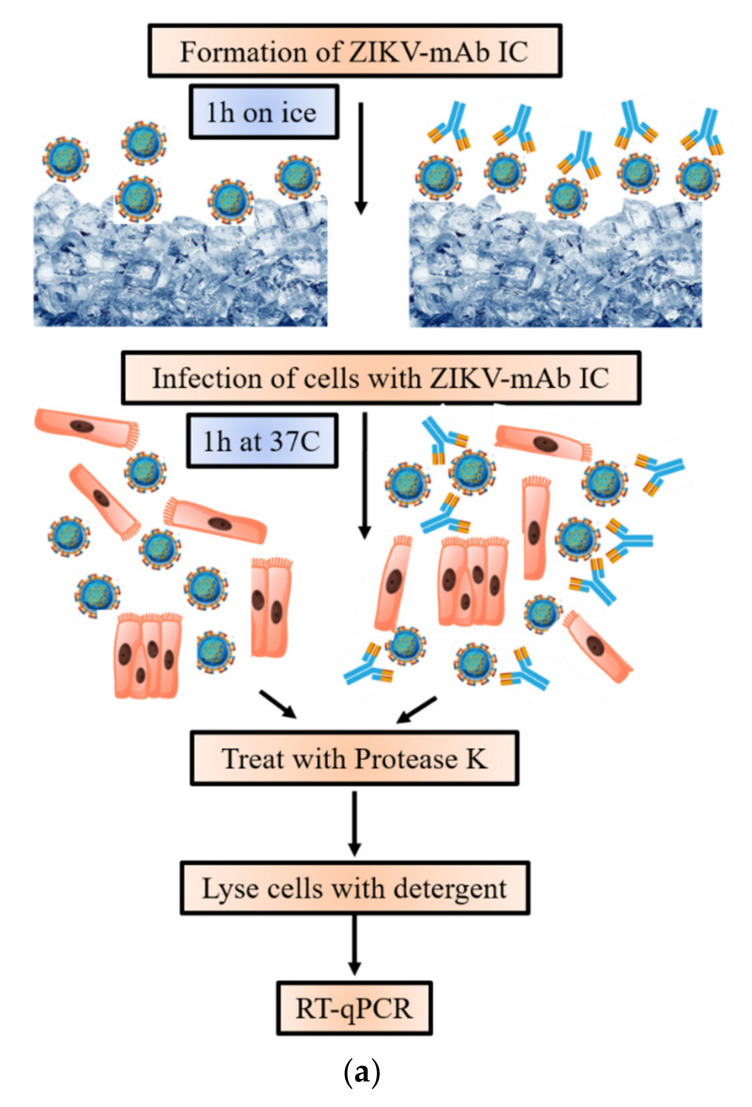

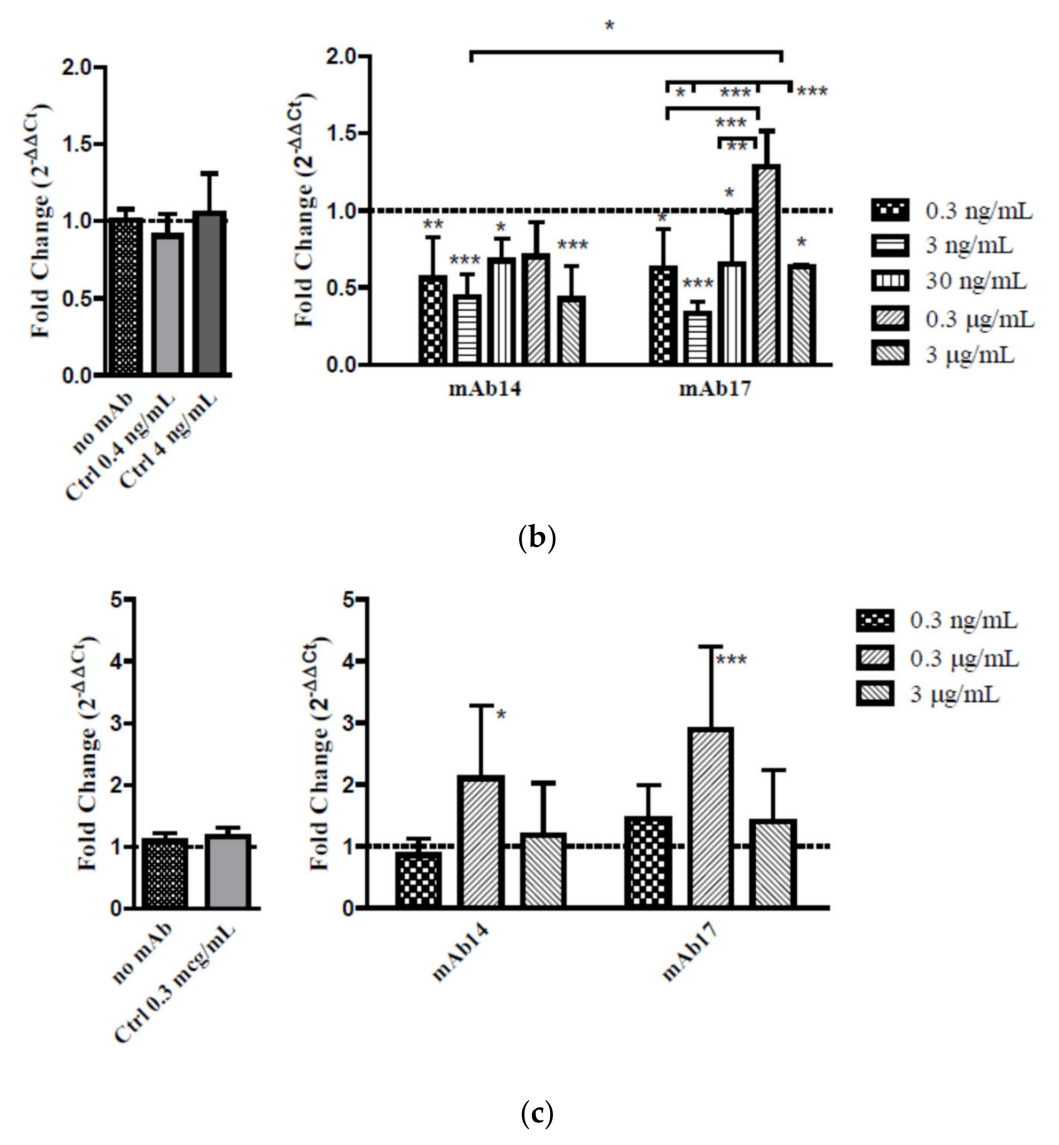
Cellular entry of Zika virus alone or as part of immune complexes (IC) in MDCK/FcRn and BeWo cells. Each column represents average value from 3–6 independent experiments; error bars represent standard deviation. (**a**) Schematic of the cell entry experiment. (**b**) Cell entry of ZIKV in MDCK/FcRn cells in the presence of anti-ZIKV mAb14 and mAb17 showed a bimodal behavior with two local minima at 3 ng/mL and 3 µg/mL. (**c**) In BeWo cells an intermediate concentration (0.3 µg/mL) anti-ZIKV mAb14 and mAb17 significantly enhanced viral entry; no change was seen at the lowest and highest concentrations tested. In both cell lines, there was no change in viral entry by unrelated IgG at similar concentrations. Data analyzed with 2-way ANOVA with Bonferroni correction; significance shown above each bar for the comparison to the no-mAb control group; * *p* < 0.05, ** *p* < 0.01, *** *p* < 0.001.

To assess cell entry of ZIKV alone or as part of immune complexes, we performed viral entry assays in the presence of either ZIKV-directed mAbs or unrelated IgG (Figure 4). For this, a suspension of 5 × 10^5^ MDCK/FcRn or BeWo cells were incubated with a large excess of virus (MOI ~100) alone or bound with IgG (schematic shown in Figure 4a). ZIKV RNA inside the cells was measured using qPCR with GAPDH as internal control. The differences in viral entry were quantified by computing relative change from the experiments with ZIKV alone. The results are shown in Figure 4b for MDCK/FcRn cells and 4c for BeWo cells.

In MDCK/FcRn cells, at low and high concentrations of IgG there was significant reduction of viral entry. The two mAbs exhibited significantly different behavior (two-way ANOVA). When compared to ZIKV alone, intermediate concentrations of IgG did not decrease (mAb14) and may have potentially increased (mAb17) viral entry. There were no significant differences in ZIKV entry among various mAb14 concentrations. The enhancement of viral entry in the presence of mAb17 compared to ZIKV alone was not statistically significant; the enhancement was significant compared to 0.3 and 3 ng/mL mAb17. No changes in viral entry were seen at similar (0.4 and 4 ng/mL) concentrations of unrelated IgG.

Similar bimodal behavior was seen in BeWo cells, with no change in viral entry compared to ZIKV alone at 0.3 ng/mL and 3 µM mAb concentrations (Figure 4c) and a statistically significant enhancement at 0.3 µg/mL mAb14 and 17. ZIKV entry in the presence of 0.3 µg/mL unrelated IgG was no different than ZIKV control and mAbs were not different from each-other by two-way ANOVA.

### 3.3. Characterization of IC

**Figure 5:**
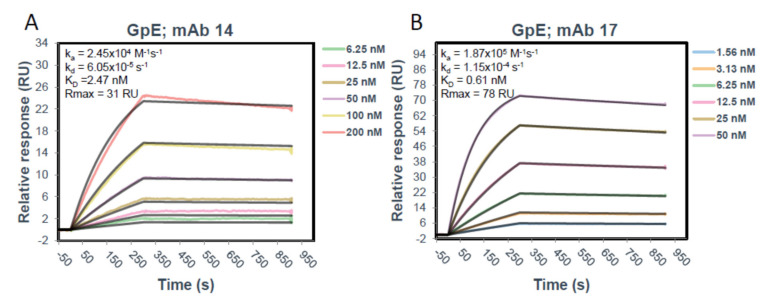
SPR sensograms showing the kinetics of gpE binding to mAb14 (**A**) and mAb17 (**B**) captured with anti-Fc antibody and the calculated kinetic constants. Under the conditions of this experiment, the antibodies bind gpE with high affinity, with mAb17 binding approximately 4 times stronger than mAb14. Additionally, the Rmax of mAb 17 is approximately twice that of mAb and theoretical Rmax possibly indicating that mAb 17 binds a gpE dimer.

The solution properties of therapeutic mAbs, especially their affinity for the immunogen and their aggregation can play an important role in antibody activity and their in vivo disposition. Using SPR we assessed the binding strength of mAb17 and mAb14 to the gpE (Figure 5). Both antibodies bound gpE with nanomolar affinity, with mAb17 having four times higher affinity (0.61 nM) than mAb14 (2.47 nM). The maximum binding capacity of gpE to mAb 17 (Rmax = 78 RU) is also approximately twice that of mAb 14 (Rmax = 31 RU), possibly indicating that mAb 17 is binding a gpE dimer.

**Figure 6:**
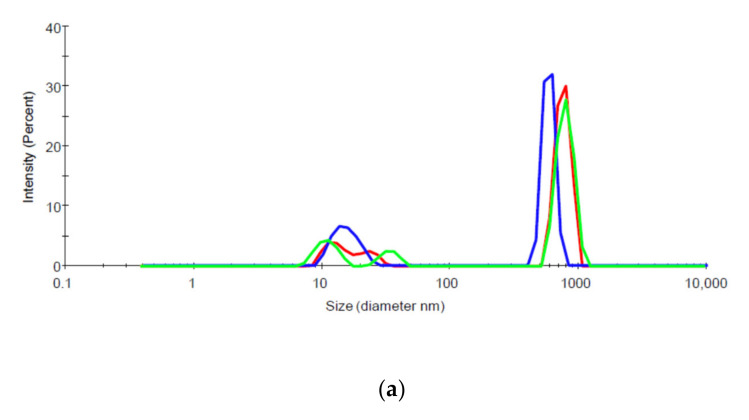

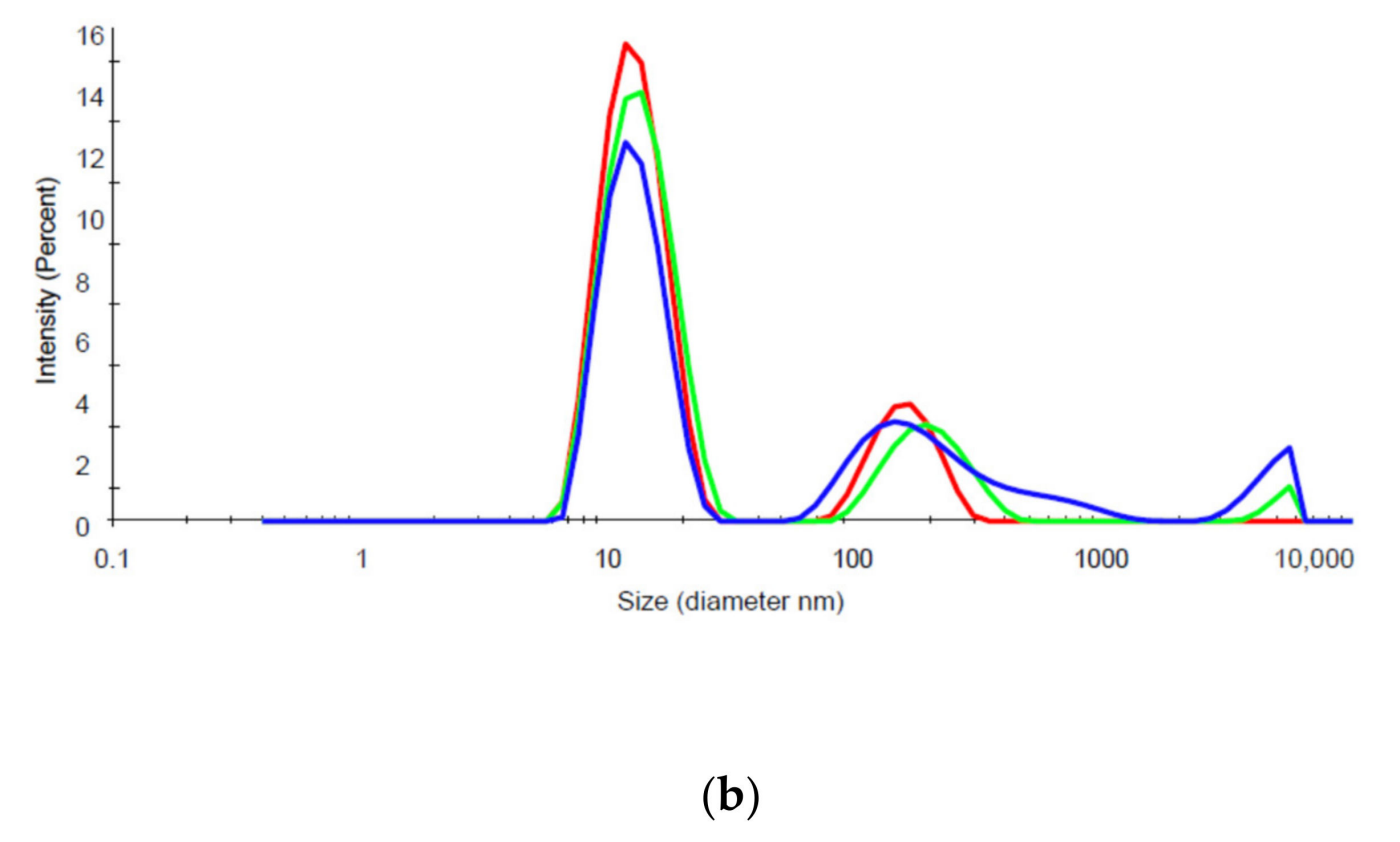
DLS traces of mAb14 (**a**), mAb17 (**b**). Different colors depict three different measurements. Larger aggregates are seen for mAb14 as indicated by the size corresponding to the major peak. The major peak from mAb17 solution appears at a position typical for IgG molecules.

Additionally, we evaluated the levels of aggregation in solution for mAb14, 17 (Figure 6a,b, respectively) and their respective IC with gpE (data not shown) using DLS. We found that, mAb14 (Figure 6a) is aggregated at room temperature, as indicated by the presence of solution particles of large hydrodynamic radius. These large aggregates are not seen in mAb17 solution (Figure 6b), where the main peak appears in a hydrodynamic radius typical for IgG molecules.

## 4. Discussion

ZIKV and other flaviviruses enter cells through attachment factors and specific receptors that mediate endocytosis of the virus and subsequent release of the viral contents into the cell [16]. Receptors include DC-SIGN, the mannose receptor, and members of the TIM and TAM family of phosphatidylserine receptors [17]. Antibodies against ZIKV can protect via various well described mechanisms, including blockage of cell entry through direct binding to envelope glycoprotein and subsequent destruction of pathogens by effector cells. However, it is possible for virus-antibody complexes to enter immune cells through various Fc receptors, be carried as cargo in endosomes, followed by endosomal acidification and membrane fusion, which permits release of viral RNA into the cytoplasm and subsequent viral replication. This mechanism has been identified as the reason for increased disease severity following Dengue virus (DENV) infections in subjects with existing immunity to DENV of a different serotype [18,19]. The phenomenon, referred to as antibody dependent enhancement (ADE), has been proposed to occur for ZIKV in vitro and in animal studies [20,21]. It is thus important that, before being tested in clinical trials, antibody preparations targeting ZIKV be assessed for their ability to block infection without the possibility of ADE. Such tests are often performed in animal models of infection and in cell lines that express Fc-gamma receptors. Although not often discussed, the FcRn, an Fc-receptor highly expressed in placenta, could also mediate ADE. We set out to test this hypothesis using a syncytium-forming human trophoblast cell line and dog kidney epithelial cells engineered to overexpress human FcRn.

We showed that ZIKV envelope glycoprotein alone or in complex with IgG can be transported through MDCK/FcRn and BeWo epithelial cells (Figure 1 and Figure 2). Although not surprising given these cells’ permissiveness to infection by ZIKV [22,23,24], to our knowledge this is the first time this observation has been made. Both cell entry and transcytosis of gpE is greater in MDCK compared to BeWo cells (Figure 1c and Figure 2e), which correlates with reduced susceptibility of the latter to ZIKV infection [24]. Intact virus was also transcytosed in MDCK cells (Figure 1d), as it has been reported for the HULEC-5a endothelial cell line [21]. It is not known if such passages through cell monolayers over a short time (transcytosis was assessed at 90 min) occurs for other viruses. Polarized entry and release of ZIKV was shown in Caco-2 cell monolayers [25], indicating that directional transport is a feature of this virus and could play a role in viral uptake and infectivity.

Given that transcytosis in MDCK/FcRn and BeWo cells occurs for both gpE and intact ZIKV, it is likely that the process is mediated (at least in part) by ZIKV receptors that directly interact with gpE. In such a case, the addition of anti-gpE IgG would compete with receptor binding and thus cause a reduction of cell entry and transcytosis. Indeed, while the addition of sub-stochiometric amounts (10 µg/mL) of mAb14 and mAb17 resulted in non-significant changes in transcytosis and cell entry of gpE (Figure 1b,c), approximate stochiometric amounts (100 µg/mL) significantly reduced entry (mAb14 and mAb17, Figure 2e) in MDCK/FcRn cells. To better address the effect of the IgG concentration on viral entry, we performed carefully controlled experiments of ZIKV entry in cell suspension (Figure 4). We diluted mAb14 and 17 over a wide range, from 0.3 ng/mL to 3 µg/mL and, for each dilution measured the effect in reducing or enhancing viral entry. These studies showed that, in cells that overexpress FcRn, a stepwise increase in IgG concentration first decreased then increased viral entry. A second reduction of viral entry was seen at a high concentration of IgG. An analogous bimodal behavior was recently described for MERS coronavirus in presence of Mersmab1.

A similar concentration dependence of ZIKV entry was seen in BeWo cells. The lowest (0.3 ng/mL) and highest (3 µg/mL) concentrations tested had little effect on viral entry, whereas intermediate concentration (0.3 µg/mL) resulted in enhanced viral entry. The lack of significant inhibition of viral entry in BeWo cells comports with lower infectivity of ZIKV in these cells [24] and likely results from lower levels of gpE-specific receptors in these cells. On the other hand, unlike in MDCK/FcRn cells, the enhancement of viral entry in BeWo cells at intermediate concentrations was statistically significant, of higher magnitude, and occurred for both mAbs.

The antibodies we used have an affinity for gpE equal to 2.5 and 0.6 nM (Figure 5), thus would be expected to bind gpE and block viral entry through gpE specific receptors even at very low concentrations. While a 50–60% reduction of viral entry at low concentrations in MDCK/FcRn cells was not surprising, it was unexpected that increasing antibody concentrations abrogated this inhibitory effect. We postulate that raising IgG concentrations would result in more immune complexes and higher cell entry via antibody Fc mediated FcRn receptor binding. This would negate some of the Fab mediated blockade of ZIKV entry. That this increased viral entry occurred at intermediate IgG concentrations would be in agreement with the µM-level affinity of the IgG for human FcRn [26,27]. At yet higher concentrations of IgG, it is conceivable that excess unbound antibodies would compete with ZIKV IC for entry through FcRn receptor, thus resulting in a second reduction of cellular entry. It is possible that other mechanisms, such as increased degradation could also be involved.

Our experiments suggest that, at specific concentrations, even high affinity IgG antibodies may not reduce and could enhance ZIKV entry in FcRn bearing cells. Such enhancement was recently demonstrated in vivo and in vitro [21]. Specifically, pre-existing DENV antibodies resulted in enhanced fetal disease in wild type mice but not FcRn^-/-^ mice. In addition, high concentrations (200–500 µg) of anti-flavivirus cross-reactive antibody together with ZIKV on the apical side of endothelial cells resulted in enhanced infection of trophoblast cells on the basolateral site. Our in vitro studies point to increased ZIKV viral entry in placental cells in the presence of an anti-flavivirus antibody (mAb17) as well as an anti-ZIKV specific monoclonal antibody, mAb14. Notably, mAb14 has been shown to enhance infection of K562 immune cells (Alpha Diagnostic International, Product Data Sheet), leading us to suspect that an Fc-mediated viral entry process underlies the enhancement in both Fc-gamma and FcRn bearing cells. Our data suggests that the concentration of cross-reacting and ZIKV-specific antibodies plays a critical role in inhibition versus viral entry, which underscores the importance of in vitro and in vivo testing of any antibody based prophylactic or therapeutic treatment. More studies are needed to better understand the process of ZIKV transcytosis, antibody mediated enhancement of viral entry and the relevance of these findings in ZIKV infection and placental transport in vitro and in vivo.

Another interesting finding emerged when we assessed disposition of the antibody element of the ICs. Increasing amounts of gpE were associated with decreased amounts of IgG transcytosis and a simultaneous depletion of the IgG in the input reservoir. This observation supports the possibility that more mAb are channeled towards degradation pathways rather than undergoing sorting and recycling as is the case for un-complexed antibodies. Furthermore, mAb14, was depleted faster than mAb17. This antibody had both lower affinity for gpE (Figure 5) and greater aggregation in solution (Figure 6). It is possible that these less favorable physicochemical properties of mAb14 play a role in its degradation.

FcRn is crucial in maintaining IgG homeostasis in circulation [6] and its blockade has been implicated in reduction of plasma IGG and autoimmune complexes [28]. It has been recently proposed that in vivo half-life of mAb correlates with transcytosis in cells that overexpress FcRn [29]. Although mechanistic studies are scarce, it is believed that the degradation of immune complexes occurs inside vacuoles, i.e., endosomes and lysosomes following pinocytosis or via receptor-mediated entry [30]. An alternative pathway could be through proteasomes, perhaps through a process mediated by TRIM21, an intra-cellular Fc receptor and ubiquitin ligase. In this pathway, the antigen/antibody complex passes through the membrane of the endosome and enters the cytoplasm [31]. In our study we observed that larger IC undergo less transcytosis and seem to be degraded faster than the un-complexed or less aggregated antibody. We postulate that an increased degradation of such larger complexes may be due to the engagement of several cellular pathways for proteolysis, for example lysosome and proteasome pathways. Larger aggregates would engage the lysosome pathway more readily and effectively than smaller ones, thus promoting a faster degradation. More studies are needed to better understand these pathways, and their significance in virus and antibody clearance in vivo.

## Figures and Tables

**Table 1:** Primers for RT qPCR.

ZIKV 1086	CCGCTGCCCAACACAAG
ZIKV 1162c	CCACTAACGTTCTTTTGCAGACAT
ZIKV 1107- FAM	AGCCTACCTTGACAAGCAGTCAGACACTCAA
Canine GAPDH Forward	GCAAAGTGGATATTGTCGCC
Canine GAPDH Reverse	TTTCCCGTTCTCAGCCTTG
Canine GAPDH Probe—FAM	TGCCGTGGGTAGAATCATACTGGAAC
Human GAPDH Forward	CCACTCCTCCACCTTTGAC
Human GAPDH Reverse	ACCCTGTTGCT GTAGCCA
Human GAPDH Probe—FAM	TTGCCCTCAACGACCACTTTGTC

## Data Availability

Datasets analyzed during the study are available from the corresponding author on reasonable request.

## Supplementary Materials

The following are available online at https://www.mdpi.com/2076-393X/9/2/145/s1, Figure S1: Complete uncropped images of the Western blots.
