# Supplementary material for: Entry and Disposition of Zika Virus Immune Complexes in a Tissue Culture Model of the Maternal-Fetal Interface
Source: Vaccines (Basel). 2021 Feb 11;9(2):145. doi: 10.3390/vaccines9020145 (PMC7916977; doi:10.3390/vaccines9020145)
Supplement: Supplementary file 1 [file vaccines-09-00145-s001.pdf]

Supplemental Figures

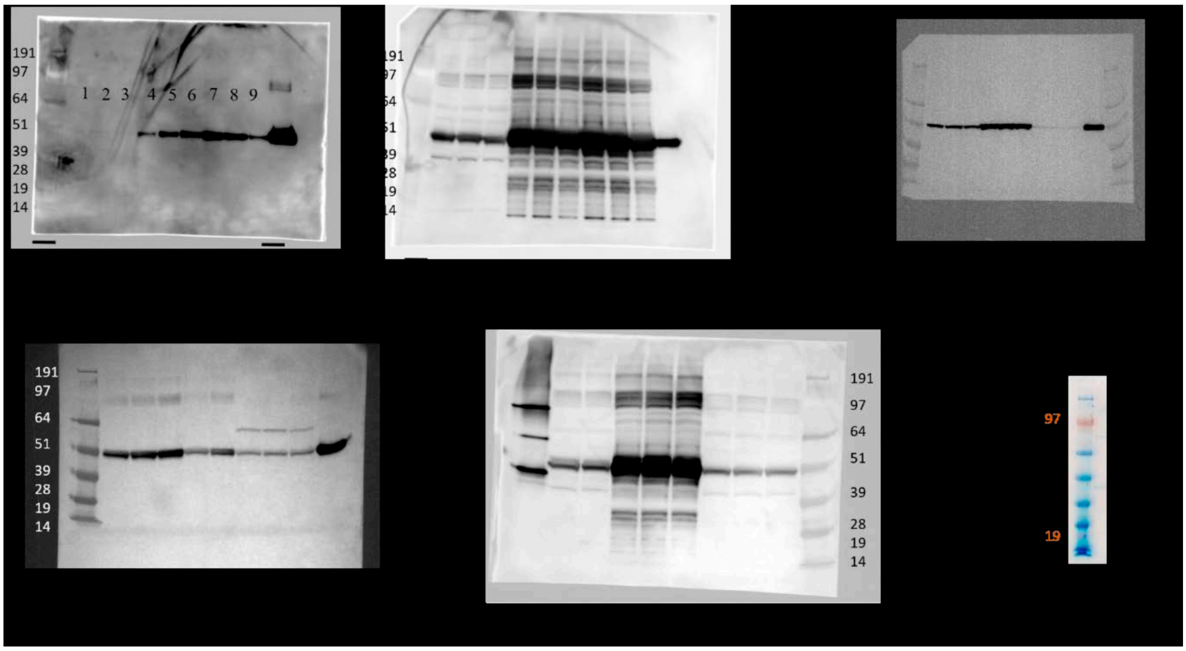

Figure S1. Complete uncropped images of the Western blots shown in text figures 1-3. The images appear in the same orientation as shown in text and are numbered with the corresponding text figure number at the bottom right corner of the image. Molecular weight markers and the positive controls are indicated. An image of an SDS gel of the pre-stained molecular weight markers is shown for reference.
